# Precautionary Principle in Adjudicating Uncertainties: A Short Review on Science and Law

**DOI:** 10.1007/s00267-026-02555-5

**Published:** 2026-07-10

**Authors:** Changdeok Gim, Nicola Ulibarri, David L. Feldman

**Affiliations:** 1 Department of Technology and Society, The State University of New York Korea, Incheon, South Korea; 2 University of California Irvine, Water UCI, USA; 3 Department of Urban Planning and Public Policy, University of California Irvine, Irvine, CA USA; 4 Water UCI, Department of Urban Planning and Public Policy, University of California Irvine, Irvine, CA USA

## Abstract

This research explores the institutional application of the “precautionary principle” to addressing scientific uncertainties, with a focus on how the international legal framework adjudicates environmental risks associated with the release of nuclear-polluted wastewater from the 2011 Fukushima Nuclear Disaster site. Scientific uncertainties tend to be amplified and escalated into political disputes due to the diversified assessments and standards on the potential risks of the discharged wastewater to public health in the East Asian region. This study asks: What institutional mechanisms can be an effective venue to manage scientific uncertainties and mitigate social amplifications of risk? This research analyzes the current legal frameworks, such as the United Nations Convention on the Law of the Sea (UNCLOS) and the World Trade Organization (WTO)’s resolution on genetically modified organism (GMO) imports to the European Union, to clarify the complexity of law and uncertainties and how they apply to the Fukushima case. Recognizing the innate gaps where technological assessment is limited, this paper suggests considering the precautionary principle as “gatekeeping” in international courts to bridge the gaps in conventional risk assessment. This approach aims to narrow methodological discrepancies between legal procedures and scientific corroboration on uncertainties, as a deliberative approach to scientific uncertainties in adjudication. Without such a mechanism, long-horizon environmental harms like those in the Fukushima case will continue to be excluded from international adjudication.

## Introduction

In courts, where the facts are institutionally constructed with scientific authority and legal procedures, the precautionary principle illuminates not a doctrinal abstraction but a knowledge translation rule grounded in co-production, one that governs how uncertainty is rendered legitimate across epistemic domains (Jasanoff, 1995, 2004, 2007; Feldman and Hanahan, 1996; Kates et al., 2001; Camacho, 2009; Feldman and Ingram, 2009; Ulibarri, 2018, 2019; Wyborn et al., 2019; Miller and Wyborn, 2020). Yet the prevailing legal frameworks for invoking the precautionary principle have not adequately addressed the epistemological character of the uncertainties they purport to adjudicate. Thus, this study’s analysis recasts adjudication on technical problems into a question of “how to translate scientific uncertainties for policymaking in courts.” The issue is not merely a boundary dispute or competing frames (e.g., shifting from the limits of predictability to a single design parameter)^1^ but an agenda of co-production of science and law (Jasanoff, 1995, 2004, 2007).

Tackling the limitations of conventional approaches to uncertainty adjudication, this paper addresses two main sets of questions intersecting law and scientific uncertainty. First, it asks how legal issues are escalated and amplified with scientific uncertainties: How are scientific uncertainties embedded in legal disputes contested, corroborated, and reconciled through legal regimes? Secondly, it explores the limitations of the international legal regime in defining and adjudicating scientific uncertainties: How has the interpretation of these legal schemes been diversified, given scientific uncertainties? Is the international legal regime effective in specifying and alleviating scientific uncertainties? Can the precautionary principle be considered in adjudicating scientific uncertainties at the international level? ^2^

To answer the research questions, this study first situates its research scope of adjudication within debates on post-normal uncertainty, which Funtowicz and Ravetz (1993) defined as epistemological uncertainties. In this context, the traditional scientific methods and technological solutions are constrained, and with limited data provided, cause confusion, not clarification, leading to conflicting conclusions depending on domains and models. Courts and tribunals confronting such post-normal conditions face a fundamental challenge. They must render legally binding judgments from uncertainties embedded in scientific knowledge that are, by their nature, provisional, contested, and irreducibly incomplete. Throughout the analysis, this paper maintains a distinction between methodological uncertainty, which concerns data limitations and modeling constraints that conventional risk assessment can accommodate through concentration-based criteria and snapshot measurement, and epistemological uncertainty, which is characterized by the irreducible incompleteness of scientific knowledge about long-horizon, cross-scale effects that resist quantification and generate confusion with traditional methods and limited data (Funtowicz and Ravetz, 1993; Stirling, 2007).

The theoretical perspectives introduced in this study are not merely presented in parallel; rather, they perform mutually complementary functions of analysis to articulate the central claim: precautionary gatekeeping. Specifically, the concept of post-normal science (Funtowicz and Ravetz, 1993) elucidates the epistemological characteristics of uncertainties at issue, while the theory of knowledge co-production (Jasanoff, 1995, 2004) provides a comprehensive lens for examining how science and law jointly and structurally construct factual knowledge out of such epistemological uncertainty within the process of judicial decision-making. In addition, risk governance scholarship (Renn et al., 2004; IRGC, 2005) illustrates how this theoretical development can be understood and interpreted as a comprehensive approach to governance of various risks and uncertainties, and ultimately plays a role in clearly theorizing the existing institutional gap in international adjudication that this study seeks to conceptualize as “precautionary gatekeeping.”

For adjudication, what counts is not a boundary dispute but the categorization of uncertainties and science-law co-production that matters in adjudication. This distinction carries direct consequences for governance, because the institutional tools appropriate for managing each type of uncertainty differ fundamentally (see Ulibarri, 2019, for scientific, administrative, and strategic uncertainties; Gim, 2026). When an epistemological uncertainty is reduced to a technical problem, for instance, the question “what are the unknowns outside our epistemic boundary to predict long-duration effects of nuclear wastewater?” can be replaced by “in what way and to what extent can we assess the risks of discharged wastewater to environments?”

To note, however, this article does not compare varying domain perspectives in the following sections because they govern the same subject matter. It compares them because they adopt different structures of translating scientific uncertainty in courts. Marine governance manages uncertainty by focusing on procedural requirements (e.g., monitoring, reporting, and cooperation) leading to international agreements, and WTO SPS jurisprudence methodologically disciplines uncertainty through risk-assessment sufficiency. Domestic gatekeeping under the Daubert line of cases, by contrast, vests courts with the authority to screen and evaluate the reliability and admissibility of scientific methodology and evidence.

The Fukushima discharge remains the empirical anchor. Each forum’s different stance on institutional design applied to scientific uncertainties exposes the institutional limitations to adjudicating Fukushima discharge. “Risk assessment, scientific uncertainties, and the adjudicative gap” identifies the adjudicative gap in the traditional technical approach to assessing risks. “The 2011 Fukushima Nuclear Disaster, contaminated water and the precautionary principle” diagnoses the limits in translating uncertainty into precaution in marine governance. “The limitations of risk assessment and possibilities for reform: WTO jurisprudence and the gatekeeping of domestic cases” then compares WTO SPS jurisprudence and domestic gatekeeping to examine alternative institutional logics for screening scientific uncertainties in international adjudication.

## Risk Assessment, Scientific Uncertainties and The Adjudicative Gap

### Scientific Uncertainties and Risk Assessment

Risk assessment can be defined as the evaluation of the likelihood of harm derived from hazards, depending on the way impacted entities are exposed to the hazards (National Research Council, 1983). Because the complexity of natural or social phenomena often involves many interlinked factors influencing the operation and outcome of systems, the formula for assessing risk has numerous unknowns, including the severity of harms, frequency of exposures, and types of hazards.

**Fig. 1:**
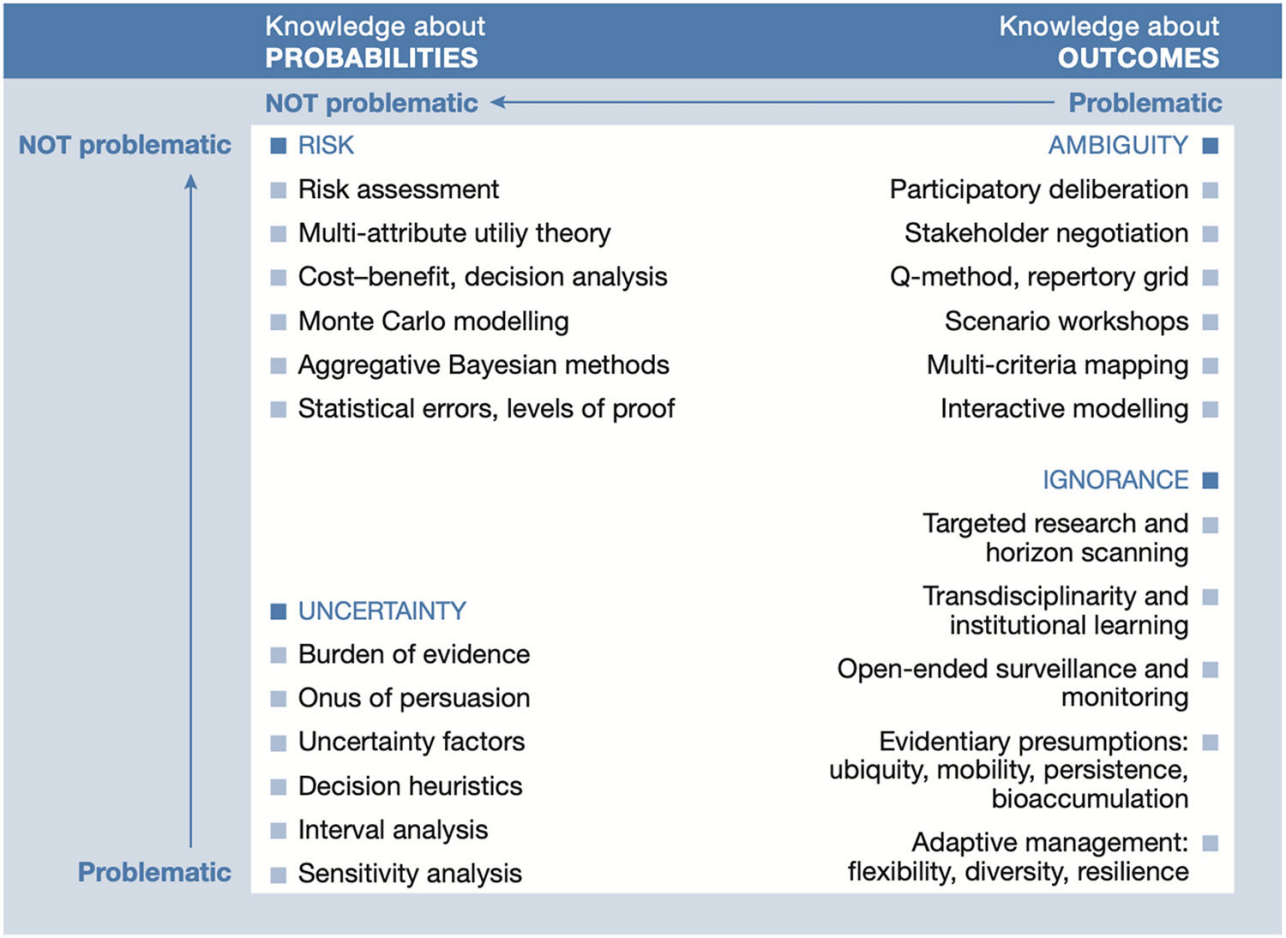
Methodological responses to different forms of uncertainty (Stirling, 2007: p. 312)

As such, the rise of scientific uncertainty issues poses a persistent and growing challenge to the frameworks of environmental governance and law (Feldman and Hanahan, 1996; Renn et al., 2004; IRGC, 2005; Stirling, 2007; Camacho, 2009; Feldman and Ingram, 2009; Ulibarri, 2018, 2019; Renn, 2026). Unlike a mere gap in information and data, uncertainty is institutionally produced, framed, and politicized through social arrangements (Jasanoff, 1990; Funtowicz and Ravetz, 1991; Sarewitz, 2004; Fisher, 2007, 2013; Hansson, 2020). In particular, scientific uncertainties, characterized by incomplete knowledge on limited data of hazards, indeterminate methodologies, and judgmental assessments, can hinder precise risk evaluations in many ways. First, obscured or questionably valid data hampers the sorting and selection of collected data (Yu et al., 2025; Akse, 2024). Second, the embedded limitations of models raise a question of whether to include or exclude relevant variables of data, to what extent (National Research Council, 1983; Oreskes et al., 1994; Saltelli et al., 2020). Lastly, the inherent unpredictability of involved social dynamics and political instability impacts the nature of decision-making, generating a structured pressure on the indeterminacy and judgment of scientific assessment (Jasanoff, 1990; Wynne, 1992; Jasanoff, 1995; Sarewitz, 2004; Gulati and Helmers, 2025). Courts serve as loci where stakeholders, policymakers, and nations convene, examine, and determine the scope and the characteristics of scientific uncertainties related to risk assessment (Jasanoff, 1995). Diverse compromises inhibit current risk assessments from resolving disputes derived from scientific uncertainties (Stirling, 2007) (Fig. 1).

Given limitations on time, resources, and data, the adjudications of courts and conventional risk assessment methods in laboratories rarely converge on an agreed-upon conclusion. The procedures of legal disputes are confined within reasonable time frames and the availability of resources contributing to the construction of facts in court (Jasanoff, 1995; Fisher et al., 2006). That said, the judicial construction of facts in courtrooms often diverges from what scientists modeled and analyzed with definite variables in the laboratory.^3^

Moreover, “The uncertainties involved in such scientific modeling render its use in litigation extremely problematic, especially in view of the judicial resistance to indirect and statistical forms of proof” (Jasanoff, 1995, p. 122). Particularly, when an issue involves risk assessment and scientific uncertainties, if the issue poses a question of adjudicative resolution regarding harms and compensation before a court, the reconstructed facts, the co-production of science and order (i.e., law and institutions) (Wyborn et al., 2019; Miller and Wyborn, 2020), in court can raise doubts from opposing stakeholders relying on certain assessment results from a designated group of scientists (Kanhanga, 2019; Mauerhofer, 2019). In this case, a judge’s political orientation or reasoning may amplify the skepticism from interested parties (Adelman and Glicksman, 2018).^4^

### Scientific Uncertainties and the Adjudicative Gap

With methodological means limited, scientific uncertainties are often escalated due to courts’ innate adjudicating practices. A fundamental, doctrinal difference between science and law is the discipline that is used to confirm a fact. In the science community, facts are statistically corroborated and reviewed through a peer-review system (requiring, in many cases, 95% confidence level); however, in courts, a (constructed) legal truth is dependent on a majority’s decision (i.e., over 50%) (e.g., a jury’s verdict), which is based on documents submitted by parties. The different practices and principles of the two domains, in some cases, exacerbate the possibilities of conflicts between different social entities or groups.

Therefore, in courts, one party might point out the other entity’s methodologies, leading to their argument as not fully scientific, and the other can defend their methodologies as being scientific enough to substantiate an adjudicated conclusion (Daubert v. Merrell Dow Pharmaceuticals, Inc., 509 U.S. 579 (1993); Jasanoff, 1995, p. 17). When legal regimes unilaterally reframe scientific uncertainty as a bounded deficit of data or methodologies to be resolved through further data collection or improved risk assessment methodologies (Porter, 1995; Park et al., 2013; Linkov et al., 2014), judicial translation work is systematically under the pressure of converting open-ended epistemological questions on the edge of current predictive knowledge into narrower technical or procedural questions in a way that existing institutional settings may handle, which somehow confuses both adversarial entities (Ulibarri, 2019).

In this regard, the action arena where this paper tests the above-mentioned research question and adjudicative gap is the discharge dispute around polluted wastewater from the contaminated site of the Fukushima Daiichi Nuclear Power Plant in Japan, as a case study to identify embedded uncertainties and the limitations of current legal methods to adjudicate uncertainties. Japan planned to release 1.25 million tons of treated wastewater from the contaminated area where the Fukushima Daiichi Nuclear Power Plant is located (as of 2022). Tons of water are still being used to cool down the melted-down nuclear fuel of the plant. The impossibility of treating the tritium in the contaminated water with state-of-the-art technologies (i.e., Advanced Liquid Processing System, hereafter ALPS) (McCurry, 2013; Takahashi, 2019) provides legitimacy for the Japanese government’s decision on release, since their release is regarded as the most reasonable method, even with the consideration of the environmental and public health impacts of the discharge.^5^ ^6^ Here, the contextual legal issues associated with the Japanese government’s decision are complicated by two factors: first, as observed in other policy cases (e.g., GMO regulation), data on scientific uncertainties have not been clarified to be reviewed based on a unified standard by independent investigators, including the reconstruction of latent impacts years after an event (Feldman and Hanahan, 1996). Second, various courts’ decisions, given a variety of reasonable pathways, can be drawn by differing interpretations with a compromised time frame, unclear data, and limited resources.

Then, the technical question transitions to the adjudicative question. In other words, the question is not whether further concentration data can be sufficiently secured but whether the very assessment framework and methods on the concentration level of tritium-based compliance can appropriately reflect the concerns over the long-horizon, cumulative, and uncertain dispersed damages to human bodies and ecological systems. The failure to identify the uncertainty characteristics differentiated from technical and methodological uncertainties has challenged international tribunals in warranting the adoption of precautionary principles. Given the limited availability of methods, data, and resources, can an international forum determine whether the precautionary principle justifies restricting the discharge of ALPS-treated wastewater from Fukushima?

Jasanoff (1995, 2004, 2007) has maintained that neither science nor law can independently resolve the epistemological uncertainties that arise in legal proceedings, highlighting instead a process of co-production through which scientific and legal knowledge are mutually constituted. Responding to the challenge of uncertainty, domestic courts in the United States have developed a more flexible approach through judicial gatekeeping in regard to the admissibility and reliability of scientific evidence (see “The limitations of risk assessment and possibilities for reform: WTO jurisprudence and the gatekeeping of domestic cases”). In this paper, we illustrate varying perspectives of social and institutional limitations as well as the possibilities of the application of the precautionary principle to the Japanese government’s discharge of radioactive wastewater, rather than to the scientific evidence itself.

## The 2011 Fukushima Nuclear Disaster, Contaminated Water and The Precautionary Principle

### Background

The Fukushima ALPS-treated water discharge case illustrates how legal framing interacts with persistent scientific indeterminacy. The key point is that institutions must determine whether uncertainty is a reason to proceed under technological safeguards or a reason to pause under precaution. Establishing this baseline clarifies what the later comparisons diagnose. What standards do legal venues adopt to translate uncertainty into an adjudicative decision?

On 11 March 2011, the 9.0 magnitude Tohoku Earthquake and the unprecedented tsunami disrupted the electricity supply system for the Fukushima Daiichi Nuclear Power Plant and inundated emergency generators. These technological failures caused a subsequent failure of the cooling system of the power plant. The station blackout and the cooling system shutdown on March 11th gave rise to the disastrous core meltdown. The Japanese government immediately responded to evacuate residents and avoid a complete reactor meltdown. However, the death toll was over 15,000 people from the tsunami, and impacted communities were devastated by the flooding and radioactive debris (Funabashi and Kitazawa, 2012; Nagataki et al., 2013). Even worse, the melted reactors 1, 2, and 3 emitted radioactive materials into the atmospheric environment after the disaster and required 150 tons of contaminated water per day to cool down the remaining nuclear fuels at the site as of 2022. Since the earthquake and reactor meltdown in 2011, groundwater contaminated with radionuclides has flowed from the affected area into the Pacific Ocean after contacting the fuels and nuclear wastes. The contaminated water has leaked from the cracks and holes of the reactor buildings and has polluted the groundwater and oceanic water adjacent to the damaged area (Takahashi, 2019).^7^ Since they found the leakage, the Japanese government and Tokyo Electric Power Company (TEPCO) have blocked and stored the leaking contaminated water, which otherwise would have seeped into groundwater and the Pacific.

Embedded uncertainties in the contaminated water are recognized by the IAEA investigative organization.^8^ Even though the IAEA report (2022) confirmed, according to TEPCO’s submitted report, that the concentration of diluted water would minimally affect “the assumed representative person,” the IAEA report (2022) simultaneously emphasized that a “further evaluation” is necessary.^9^ Also, IAEA noted that all supporting data and underlying methodologies for TEPCO’s report should be made available to the public for the accountability of the assessment procedures and results.^10^ The IAEA’s Comprehensive Report (2023) found that Japan’s discharge method meets international safety standards from concentration-based measurement and that the expected radiological impact is minimal. However, the IAEA Task Force review (2022) also noted that a more nuanced approach to source term characterization is needed (p. 36) and recommended strengthening long-term monitoring to mitigate uncertainty (p. 45). Recent scientific literature lends credence to these concerns (see Appendix C). The absence of long-term evidence of accumulated damages is, in itself, an epistemological uncertainty that highlights a critical gap in the international adjudicative framework’s institutional capacity to handle such cases.

### Wastewater Discharge from the Damaged Site and International Marine Governance

In the marine-governance regime for the land-to-sea discharges, monitoring and concentration thresholds become proxies for legality even when they cannot resolve long-horizon environmental uncertainties. This gap motivates the comparative move. What happens when a forum translates uncertainty disputes into risk-assessment adequacy and evidentiary sufficiency?

This section discusses the current regime for regulating the discharge of polluted wastewater from land into the ocean and examines the extent to which existing legal and institutional arrangements can effectively govern the international discharge of radiologically polluted water to the ocean. According to Article 1.1. (i), the United Nations Convention on the Law of the Sea (UNCLOS) excludes the Japanese government’s discharge of nuclear-polluted wastewater from the definition of “dumping,” which is defined as “(i) any deliberate disposal of wastes or other matter from vessels, aircraft, platforms or other man-made structures at sea.” In this case, the water is not discharged from “man-made structures” at sea^11^ but through pipelines from the impacted site.^12^

Recognizing that international nuclear regulation^13^ and international customary law of transboundary environmental harm^14^ are not applicable, then, the issue here is whether or on what conditions “necessary further evaluation” to assess risks (e.g., article 206, international assessment) based on the suggestion of the IAEA report can appropriately be applied at the international level despite the provisional duties of protection of the marine environment. For instance, according to article 207(1), “states shall adopt laws and regulations to prevent, reduce and control pollution of the marine environment from land-based sources, including (…) pipelines and outfall structures, taking into account internationally agreed rules (…).” Also, “states shall endeavor to harmonize their policies in this connection at the appropriate regional level.”^15^ Accordingly, and pursuant to article 206, states shall, “as far as practicable, assess the potential of such activities on the marine environment and shall communicate reports of the results of such assessment in the manner provided in article 205.”^16^

Despite plural provisions on arranging conditions for the discharge from the “site,” any provision is not applicable to preventing the discharge itself as long as the Japanese government abides by the international nuclear waste concentration standard according to Article 207(1).^17^

Within this marine-governance regime, scientific uncertainties about long-horizon effects are institutionally normalized, and procedural compliance substitutes for substantive thresholds of harm. The ITLOS Advisory Opinion in Case No. 31, though addressed to greenhouse-gas-induced marine pollution, reinforces this translation gap and supports a precautionary reading of such uncertainty. In particular, the Advisory Opinion (Case No. 31, paras. 212-213) rejected the coupling of scientific assessment and the institutional control over pollutant substances in environments and weighed in on “other relevant factors” that constitute “the best available science” (see Appendix C for the extended analysis). The following sections turn to WTO SPS jurisprudence and domestic gatekeeping to examine whether they offer what marine governance cannot.

## The Limitations of Risk Assessment and Possibilities for Reform: WTO Jurisprudence and The Gatekeeping of Domestic Cases

Given the uncertainties of long-term effects of exposure to low doses of tritium (International Atomic Energy Agency (IAEA), 2022: 36) in the case of Fukushima nuclear wastewater discharge,^18^ it is prudent to adopt proactive prevention measures at the outset. Because marine law does not provide clear guidance, we turn to the application of the precautionary principle in other legal domains in search of possible approaches. The following sections examine whether each adjudicative forum handles epistemological uncertainty in methodological terms, or recognizes it as a normative framework, or adopts a particular procedural mechanism to adjudicate it.

However, the legal doctrine on the precautionary principle still remains contested. Scholars disagree on whether the principle provides operational guidance in adjudication (Majone, 2002; Sunstein, 2003; cf. de Sadeleer, 2002; Fisher, 2007, 2013; Renn et al., 2004; IRGC, 2005; Trouwborst, 2002, 2009; European Parliamentary Research Service, 2015; Persson, 2016; Hansson, 2020; Renn, 2026). This institutional question has gained new attention following *Loper Bright Enterprises v. Raimondo*,^19^ which overturned the *Chevron* deference doctrine^20^ and shifted interpretive authority over statutory ambiguity from administrative agencies back to courts. This overturn amplifies the salience of courts’ gatekeeping, which we find absent in international forums and present in domestic adjudication.

The following sections diagnose this difference across WTO jurisprudence, customary international law, and US case law. Throughout this analysis, the Fukushima discharge serves as the empirical reference point, illustrating how each forum’s decisions would shape the adjudicative precaution on the epistemological uncertainty of long-horizon, low-dose radiological risks.

### Backgrounds

The analysis of the WTO dispute examines the methodological constraints in applying the “precautionary approach” to scientific uncertainties surrounding Genetically Modified Organisms (GMOs). By narrowing the diagnostic scope, the trade forum contrasts risk-assessment obligations with a “precautionary approach.” The GMO case and the Fukushima discharge controversy share common analytical terrain. What counts as a critical ingredient of “science”? Persistent disagreements, competing models, and divergent results structurally pervade both cases. The WTO forum translates this complexity into tractable technical questions of “methodological sufficiency” and “evidentiary burden.”

In May 2003, the United States, Canada, and Argentina filed a lawsuit against the European Union, claiming that the EU’s general moratorium and the approval delay violated the General Agreement on Tariffs and Trade (GATT). In the claim, the issues were categorized into three pieces. The first would be the General (de facto) Moratorium issue. From 1998 to 2003 there was no GM product release in the market of the EU.^21^ The issue was whether there was a violation of the WTO Agreement on the Application of Sanitary and Phytosanitary Measures (the SPS Agreement), Annex C(1)(a). They claimed that the EU’s moratorium on the approval process and the product-specific delays (i.e., “delays in approving 24 out of 27 products”) fell within the violation of “undue delay” (SPS Agreement article 8 and Annex(C)) and halted the approval processes in a manner less favorable towards imported products.

Furthermore, the US, Canada, and Argentina argued that the EU’s national ban, where six EC members put “safeguard” prohibition on approved GMO products and food, violated the SPS Agreement articles 5.1 and 5.7. In Article 5, the SPS Agreement requires risk assessment and regulates a guideline for responses to a lack of scientific data and information.^22^ In this regard, the third issue, “provisional measures” of respective states^23^ (i.e., Austria, France, Germany, Greece, Italy, Luxembourg) associated with the national bans provides more significant implications in adjudicating scientific uncertainties in relation to the Japanese government’s discharge.

### Adjudicating GMO Uncertainties with Risk Assessment

To analyze the risk assessment standards applied to the Fukushima case, this section investigates the SPS legality, specifically whether the framework allows for a precautionary approach beyond the standard of “risk assessment sufficiency” when adjudicating the epistemological uncertainties of long-horizon, low-dose radiological exposure.

The SPS legality illustrates the “risk assessment” obligations channel contested uncertainties into a technical test on whether the assessment conformed to international standards established by epistemic communities. Precautionary arguments were thus rerouted into a narrower dispute over whether the right technical methods and standards were applied. This channeling forecloses the potential of co-production of science and law and reduces disputes to whether institutionally standardized risk-assessment requirements have been satisfied. For controversial uncertainties involving long-horizon ecological harms, the effect is adjudication locked into a snapshot of technical compliance, hampering judicial efforts of adaptive co-production.

According to the WTO panel report, if there is sufficient scientific evidence, risk assessment^24^ is required,^25^ and furthermore, the methodologies of risk assessment should be developed by “international organizations” (SPS Agreement article 5.1^26^) (i.e., the Codex Alimentarius Commission).^27^ International standards should be included prior to risk assessment on “risks to human, animal or plant life or health,” which means a domestic regulatory body cannot be an entity in charge of risk assessment and implementation. Only after a decision by Article 5.1 assessment on whether there is sufficient scientific evidence, “provisional measures” based on Article 5.7^28^ can be applied (Henckels, 2006: 294). According to the SPS agreement article 5.1, 5.7 and Annex C Article 1(a), the EU’s ban on GMOs violated the doctrine of “undue delay” since there were no reliable results of “risks to human, animal or plant life or health” from an appropriate risk assessment.

Regarding legal disputes, the Panel of the WTO concluded “Arts. 5.1 and 2.2 were applicable” (the WTO panel report). In the case of “insufficient information,” the SPS agreement Article 5.7 provides a benchmark on what would be an appropriate risk assessment. According to Article 5.7, if there is any available “pertinent” information, which is derived from either “*international organizations”* or other Members’ applications, that information needs to be employed in risk assessment. Furthermore, in case of “insufficient scientific evidence,” provisional measures are needed to suffice conditions such as (i) insufficient “relevant scientific information,” (ii) “available pertinent information,” (iii) “additional information necessary for a more objective assessment of risk,” (iv) “within a reasonable period of time.” However, according to the WTO report, “[I]n no case, was the situation one in which the Panel had been persuaded that the relevant scientific evidence was insufficient to perform risk assessment (…).”^29^

In the case of the Fukushima discharge, if the WTO framework were applied, the adjudicative question would be confined to whether TEPCO’s risk assessment conformed to international concentration standards, without adequately addressing cumulative, long-horizon environmental harms.

### Different Views on the Precautionary Principle and Risk Assessment

Applied to the Fukushima discharge case, the regime of risk assessment (e.g., WTO) would require neighboring countries challenging the discharge to demonstrate, through quantitative risk assessment, that the tritium at the discharged concentration poses a sufficient risk to justify regulatory measures. However, IAEA standards and reports have already acknowledged the need for a more persuasive approach to uncertainty (IAEA, 2022, p. 36), which signifies an epistemological gap that the mere sufficiency of risk assessments cannot close.

In this regard, while the precautionary principle carries political weight, particularly in the EU’s regulatory tradition, the WTO Appellate Body (in *EC-Hormones*) has consistently declined to grant independent legal force to the “precautionary approach” within the SPS framework. The principle has been acknowledged but operationally constrained. The adjudication standard reverts to “risk-assessment sufficiency” under Articles 5.1 and 5.7. In spite of the fact that, in the European Union, the precautionary principle is critical to environmental policy and law (Foster, 2011: 20), whether Article 5.7 of the SPS Agreement suggests a different type of “precautionary principle” is clouded with a range of adjudications of the WTO Appellate Body.^30^ In the case of GMOs, the WTO Panel reserved judgment on the characteristic of “precautionary principle” as international customary law (Foster, 2011). In the GMO disputes, the WTO Panel (EC-Biotech, 2006), the Precautionary Principle was not adopted, and the United States and Canada raised a fundamental question on the assessment methodology and contested the legitimacy of scientific assessment conducted on the uncertain risks of GMO products by the EU community. The WTO Panel stated that it did not see any “need separately to examine the European Communities’ argument that these measures are based on the precautionary principle” since the Panel already examined the compliance of Article 5.7 requirements regarding provisional measures (WTO, 2006: 7.3220, 1009). Even though the WTO Appellate Body, in the *EC*-*Hormones* case, stated that “Article 5.7 reflects this precautionary principle,” neither the Appellate Body nor the EC-Biotech Panel has explicitly endorsed the precautionary “approach” as a “principle” or customary law underpinning Article 5.7. Rather, the WTO Appellate Body confirmed that the “precautionary approach” should also comply with the requirements of a preliminary risk assessment in Article 5.1.^31^

Thus, the precautionary principle cannot play a role as an exception to Article 5.1 of the SPS Agreement and only provides a means to understand the SPS Agreement. In the SPS Agreement, scientific uncertainties addressed by “precautionary principle” are differentiated from the “insufficient scientific evidence (Foster, 2009: 52).” If scientific evidence is enough, risk assessment is necessary for SPS measures, but otherwise, interim approaches are allowed for further information gathering for risk assessment. This conditional approach is based on the insufficiency of information (Foster, 2009: 51), not based on scientific uncertainties and precaution. Any additional “pertinent information” for risk assessment is still needed to justify provisional measures “within a reasonable period of time.” Arguably, as discussed in the following section, the WTO Appellate Body’s statement, thus, discloses distinctive views from the developmental trajectory of the “precautionary principle” at the very lowest level. In other words, risk assessment defined in Annex A is provided as a guideline, and precaution has not been granted independent legal standing as a self-sufficient basis for adjudicative action.

For the Fukushima case, thus, the precautionary approach would not provide an alternative adjudicative pathway in that the institutional logic of risk assessment channeling described above remains entrenched. Any challenge would need to demonstrate methodological insufficiency of the existing risk assessment, and given that Japan’s discharge meets current concentration thresholds, long-horizon ecological concerns would fall outside adjudicative review. If the precautionary approach lacks independent standing within the SPS framework’s risk-assessment requirements of Articles 5.1 and 5.7, the question is whether precaution can be recognized as a norm of customary international law, as discussed in the next subsection.

### Precautionary Principle As A Customary Law in the Evolutionary Course of Disputes

Given that the SPS framework has operationally constrained the precautionary approach (“Different views on the precautionary principle and risk assessment”), the analysis confirms that precaution remains contested not only politically but doctrinally. Despite a growing body of international instruments endorsing precaution, adjudicative bodies have declined to recognize it as customary law capable of independently triggering protective action in the face of epistemological uncertainty, leaving uncertainty disputes to be addressed reactively only after harms have materialized.

According to Article 31(3)(c) of the Vienna Convention on the Law of Treaties (1969, in force 1980), “any relevant rules of international law applicable in the relations between the parties” need to be applied in arranging disputes. The EC argued for the precautionary principle as a customary law based on international legal schemes and domestic EU Directives in the adjudication of GMO ban in Europe.^32^ Principle 15 of the Rio Declaration on Environment and Development (1992)^33^ stated that the precautionary principle is the major principle to adjudicate scientific uncertainties. Despite the lack of full scientific certainty, cost-effective measures should not be postponed in order to prevent “irreversible environmental degradation.”^34^ Further, the Convention on Biological Diversity (1993) accentuates “the need for and modalities of a protocol” to safely transfer, handle, and use “any living modified organism,” which may have the potential to degrade biological diversity. Subsequently, Article 1 of the Cartagena Protocol on Biosafety to the Convention on Biological Diversity (2003) provided “the precautionary approach” as a fundamental guideline for regulating “living modified organisms” of modern biotechnology.^35^ Article 11 of the protocol explicitly states that scientific uncertainties cannot prevent any appropriate actions from regulating the import, direct usage, and processing of “living modified organism” to reduce adverse impacts on biodiversity. However, in the interpretation of Article 5.1, the international adjudicative body rejects the role of the precautionary principle in interpreting Article 5.7 and refuses to confirm the notion of customary law in examining the precautionary principle.

Moreover, the sequential risk assessment guidelines of the EU demonstrate a perspective on risk assessment different from that of the WTO guidelines.^36^ According to Article 14 and Article 15 of the Cartagena Protocol on Biosafety (2003), the domestic standard for risk assessment would be enough as long as the method is conducted “in a scientifically sound manner.”^37^ In addition, Article 15 states that risk assessment should be conducted by the importer; however, the exporter can also be a responsible entity in charge of scientific risk assessment. Pursuant to the Convention on Biological Diversity (1993) and the following Cartagena Protocol on Biosafety to the Convention on Biological Diversity (2003), the EU Directive 2001/18^38^ provides a general obligation principle in Article 1 (objective)^39^ and Article 4 (general obligation), which emphasizes the “precautionary principle” to regulate the release and the marketization of GMOs. Annex II of the EU Directive 2001/18 defined “principles for the environmental risk assessment” but has no specific terms on risk assessments developed by international organizations.^40^ Furthermore, a more specified ruling on GMOs, namely“the Regulation (EC) No 1829/ 2003” provides a method for safety assessment which follows a “community procedure” rather than unified, international-level assessments.^41^

The status of the precautionary principle as a general principle of international law is still in dispute (European Parliamentary Research Service, 2015). For its advocates, it is a customary norm (Trouwborst, 2002, 2009)^42^ and a science-based “bypass route” backed by “scientifically valid indications of danger” (Hansson, 2020, p. 249)^43^; for its critics, the principle can be arbitrary in adjudication (Majone, 2002; Pesendorfer, 2011): “it offers no guidance” (Sunstein, 2003, p.16). Scholars’ characterizations range from a non-binding guideline^44^ under politics (Gilland, 2000) or loss aversion heuristics focusing on trade-offs between opportunities and risks (Sunstein, 2003)^45^ to a norm in the process of crystallizing^46^, to “attained the status of customary international law” (Trouwborst, 2009, p. 108; similarly, Doan, 2025). The WTO adjudicative body’s refusal to grant independent legal standing to precaution in the GMO dispute epitomizes this doctrinal divergence. The “bypass route” (Hansson, 2020) was not permitted; a deliberate institutional choice favored a narrower pathway requiring risk assessment grounded in full scientific proof. Plausible but uncertain dangers alone could not sustain regulatory action absent demonstrated scientific sufficiency (Hansson, 2020).

The “bypass route” is foreclosed as a doctrinal approach because the current institutional framework confines disputes to the narrower question of methodological insufficiency, leaving long-horizon harms outside the scope of adjudication. The analysis in the following section examines the epistemological foundations and knowledge governance on which risk assessment rests in the Fukushima case. For states seeking to challenge the Fukushima discharge on precautionary grounds, this foreclosure (of the bypass route) has a more direct impact regarding adjudicative determinations. In other words, if the precautionary principle lacks independent legal status, it cannot serve as a basis for requiring Japan to address long-horizon uncertainties that fall outside concentration-based regulatory compliance criteria.

### The Limitation of Reductionism and the Potential of Precaution

In “Different views on the precautionary principle and risk assessment” and “Precautionary principle as a customary law in the evolutionary course of disputes”, we discussed the lack of legal force of the precautionary approach within the SPS framework and as customary international law, leaving disputes to be addressed reactively. The focal discussion should be to build meaningful “inferential bridges” in courts between contested science and coordinated knowledge co-production (Jasanoff, 2004). The critical area to explore, related to precaution, is not contesting the certainty of the harmful effects of long latency with risk assessment, but the review and validation of scientific uncertainties with the “best available science.” Without pretending uncertainty has been diminished, the task should be centered on the exclusion of pressuring with reductionism and short-horizon risk metrics. The precaution is addressed as the institutionalization of procedures rather than a prescriptive outcome.

The EU’s approach to precaution and risk assessment can be better understood from the vantage of diversified risk definitions and the need ultimately to rely on professional judgment. It is evident that, as NRC pointed out, the inherent uncertainty of the traditional formula of risk (probability * magnitude of harms) is manifested in two ways: “missing or ambiguous information on a particular substance and gaps in current scientific theory (National Research Council, 1983: 28).” According to the WTO panel report, the possibility of uncertainties and value judgment has no space in understanding Article 5.7. However, intrinsically, regarding scientific modeling, components such as “inferential bridges” need to be incorporated (National Research Council, 1983: 28).^47^ Thus, in risk assessment, by definition, authorities’ judgments come to the fore in adjudicating the boundary of acceptable risks.

More broadly, the precautionary principle hinges on the limitations and humility of science (Stirling, 2007: 313) rather than the reducibility of scientific or technological options. Thus, the precautionary principle inherently includes the non-traditional risk concept, defined as “a situation or event where something of human value (including humans themselves) is at stake and where the outcome is uncertain (Aven and Renn, 2009)”.^48^ As being congruent with the recognition of scientific uncertainties and qualitative risks, the precautionary principle is grounded in value judgment to strike a balance between irreversible harm and costs.

**Fig. 2:**
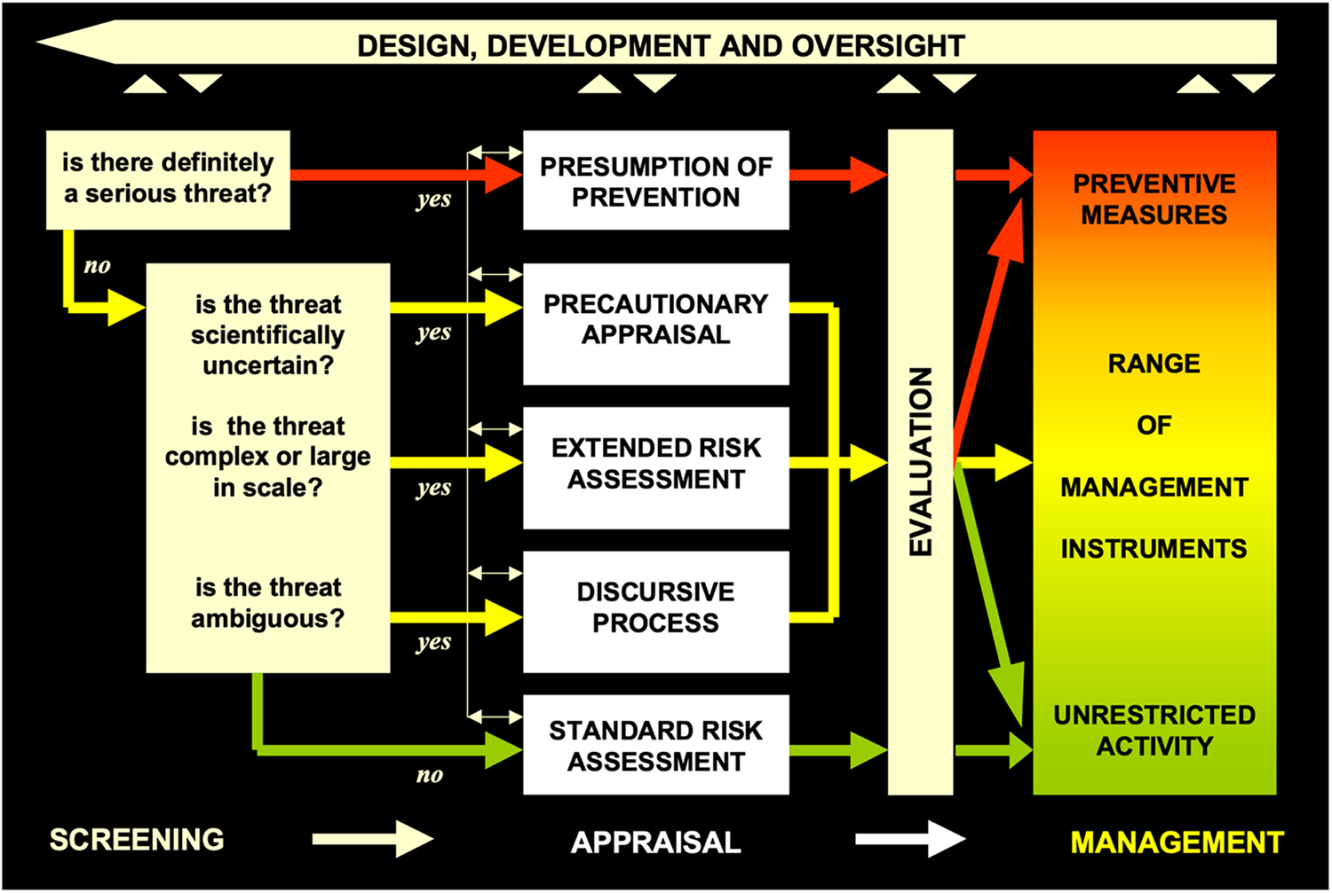
A General Model of Precautionary Risk Regulation (Renn et al. 2004, p. 6)

This reductionist limitation, the systematic reduction of epistemological uncertainties to methodological parameters, is recognized not only in the STS literature but also in legal and philosophical scholarship on precaution. Precaution and risk assessment address different sides of the same problem, two sides of the same coin: “false positives versus false negatives” (Persson, 2016, p. 139). The former operates where scientific certainty is absent, while the latter presupposes quantifiable parameters (de Sadeleer, 2002; Hansson, 2020). Ironically, Sunstein (2003)’s diagnosis of heuristic biases and trade-off neglect reinforces, rather than undermines, the procedural governance framework that Renn et al. (2004) propose for adjudicating epistemological uncertainties (Fig. 2).^49^

Fisher (2007) extends this distinction into institutional inferences, through what she terms “administrative constitutionalism,” arguing that legal frameworks addressing uncertainty should center on reviewable decision-making procedures instead of quantifiability. The “Deliberative-Constitutive” paradigm treats technological risk as “complex socio-political disputes involving values and epistemological problems” (p. 33), contrasting with the “Rational-Instrumental” paradigm that regards scientific knowledge as objective.

If international forums channel uncertainty into risk-assessment sufficiency, the question becomes how domestic courts handle the same challenge through evidentiary gatekeeping. We propose not a direct transplantation but institutional screening governance, which domestic case law offers, having developed the epistemic tools that international forums lack.

### Risk Assessment in Domestic Courts and Gatekeeping on Uncertainties

The WTO analysis reveals a recurring tension. When an international judicial framework addresses scientific uncertainty, the emphasis on risk assessment tends to frame the problem, converting irreducible epistemological questions into tests of evidentiary sufficiency. Domestic forums, by contrast, exhibit greater flexibility. Over the past three decades, the Daubert line of US case law provides a diagnostic contrast. The Daubert court actively constructs what counts as reliable scientific methodology, gatekeeping which claims of scientific uncertainty become legally viable, and which are screened out when they reach the jury. What presents itself as a scientific dispute over uncertainty has often been resolved through “inferential bridges” governing the admissibility and reliability of evidence. This contrast clarifies what is missing when international settings rely predominantly on snapshot compliance and narrower tests of scientific sufficiency, foreclosing the adaptive institutionalization of co-produced knowledge on uncertainty.

Despite the aforementioned characteristics and limitations of “scientific” risk assessments, the WTO adjudicative bodies were only concerned with “scientific” risk assessment governance in both Articles 5.1 and 5.7 and rejected the premise of scientific uncertainties as an exception to risk assessment in Article 5.7. Given that the WTO adjudicative bodies’ interpretation of “risk assessment” in Article 5.1 is based on a traditional conception, which is “the characterization of the potential adverse health effects of human exposures to environmental hazards” defined by NRC in 1983 (National Research Council, 1983: 18), the international adjudicative definition of risk firmly sticks to the combination of quantifiable variables and possibilities.^50^ Arguably, the WTO adjudicative body confined the discussion on GMO risks on public health within the boundary work between “sufficient” and “insufficient” information and evaded the discussion on the premise of *scientific uncertainties*, wherein the “inferential bridges” among optional policies are necessary.

However, in examining the relationship between law and scientific risk assessment, we need to pay attention to the fact that the determination of what constitutes admissible scientific evidence, the conditions under which it may be accepted in judicial proceedings, and the extent to which judicial decisions can be grounded in such evidence has been articulated as a judicial function of “gatekeeping,” most notably in Daubert (1993). In the Daubert case, despite the scientific community’s general standards on the acceptance of scientific evidence and assessment, the verification of scientific knowledge, who qualifies as a scientific expert, and through what procedures the expert’s testimony can be admitted are thoroughly dependent on judicial judgments. This ruling establishes that the judicial gatekeeping on what conditions scientific knowledge shapes the construction of factuality and whether evidence can be admitted as definitive scientific proof (i.e., Daubert standards: peer review, testability, general acceptance, potential error rate) is determined by the court’s judgment. (replacing Frye standard, See *Frye v. United States, 293* *F. 1013 (D.C. Cir. 1923)*)(Jasanoff, 1995, p. 17) Federal Rule of Evidence 702, as amended in 2023, and the subsequent case law further reinforce judicial gatekeeping over the admissibility of expert evidence and tort causation. (Detailed case analyses are provided in Appendix B. through *Allen v. United States*, the 2023 amendment to Rule 702, *Slatowski v. Sig Sauer*, and *Sindell v. Abbott Laboratories*.)

The recent overturn of *Chevron* deference in *Loper Bright* (2024) further reemphasizes the diagnosed trajectory in domestic law, transferring interpretive authority over statutory ambiguity from agencies to courts and thereby extending the gatekeeping approach in this research beyond evidentiary admissibility to statutory construction in adjudication. Courts’ review authority (i.e., *Loper Bright*) on the agency’s scientific judgments reinforces judicial gatekeeping over scientific uncertainties in regulation. If a Daubert-style gatekeeping inquiry were applied to the case of the Fukushima discharge, the adjudicating body would not examine whether tritium concentrations satisfy scientific consensus and regulatory thresholds. Rather, it would evaluate whether TEPCO’s risk assessment is methodologically reliable enough to account for the effects of long-term, low-dose cumulative exposure. This is the epistemological uncertainty that the IAEA itself flagged, yet remains unadjudicated under the current international framework.

## Conclusion

Synthesizing the cross-domain comparison, the central challenge from each domain is not the mere technological obstacle of scientific uncertainty but the absence of institutional mechanisms that render uncertainty admissible and constructive within adjudication (Fisher, 2007, 2013).

A cross-forum comparison yields three implications regarding how adjudicative bodies differently manage epistemological uncertainty. The WTO SPS regime reduces epistemological uncertainty to methodological uncertainty, requiring regulatory measures to be grounded in quantitative risk assessments; the problem, however, is that in the absence of scientific consensus or the presence of inherent fundamental uncertainty, this regime struggles to provide any meaningful adjudicatory guidance (“Adjudicating GMO uncertainties with risk assessment” and “Different views on the precautionary principle and risk assessment”). In contrast, arguments based on customary international law acknowledge that epistemological uncertainty itself can sometimes serve as a legitimate basis for regulatory measures, thereby challenging the blind spots inherent in the quantitative risk assessments relied upon by traditional regimes. However, this normative approach faces institutional constraints, as neither the WTO Appellate Body nor the ICJ has granted the precautionary principle sufficient independent legal status (“Precautionary principle as a customary law in the evolutionary course of disputes”). Finally, the Daubert jurisprudence bridges this gap between norm and institution. Judges, acting as gatekeepers, do not require scientific certainty; instead, when the science itself is contested, the doctrine empowers them to evaluate whether the methodology underlying a scientific claim is sufficiently reliable to construct “inferential bridges” between uncertain evidence and legal judgment.

Thus, departing from the limitations of existing legal doctrines, this paper defines precautionary gatekeeping as a structured adjudicative screening through which international tribunals evaluate, at the evidentiary admissibility stage, whether the scientific methodology addressing epistemological uncertainties (e.g., cumulative effects of low dose and long-term exposure) at issue is sufficiently reliable and adequate despite the absence of scientific certainty and definitive proof of harm. In risk assessments, the standard of “due diligence” reviews whether a state has sufficiently complied with prescribed procedures, and the “best available science” utilized as an instrumental tool relies on the existing body of scientific evidence and established methodologies as its benchmark. Precautionary gatekeeping operates where such consensus and judicial benchmarks are absent. Instead of allowing judges or adjudicators to rely on procedural compliance or wait indefinitely for definitive scientific proof, it empowers them to acknowledge and scrutinize the inherent epistemological limits of uncertain claims at the evidentiary admissibility stage.

Specifically, this screening function could be exercised at the early stage of preliminary admissibility in judicial proceedings by international adjudicatory bodies such as the International Tribunal for the Law of the Sea (ITLOS), the International Court of Justice (ICJ), or dispute settlement panels of the World Trade Organization, where necessary, with the assistance of scientific experts appointed by the tribunal. In other words, before assessing on the merits whether the risk assessment at issue satisfies substantive regulatory thresholds, the adjudicating body would first screen whether the scientific methodology underlying that assessment is sufficiently reliable to account for the type of epistemological uncertainty in question. The advisory opinion in ITLOS Case No. 31 (paras. 212–213), as discussed in “The 2011 Fukushima Nuclear Disaster, contaminated water and the precautionary principle”, which held that the concept of marine pollution inherently incorporates a precautionary approach, provides a judicial precedent supporting this institutional logic.

The Fukushima case makes this institutional gap concrete. The current international legal framework, including UNCLOS, the London Convention/Protocol, and international nuclear treaties, relies on concentration-based criteria (i.e., a short-term snapshot of measurement in time) to determine compensation for ex-post harms rather than to institutionalize precaution through ex-ante screening of technological methods entailing epistemological uncertainty.

The challenge raised in adjudicating the discharge by Japan is interrogating the status of precaution at the international level and the inconsistencies in regulation and law across different risk-policy domains. Given the analysis on international environmental regimes to regulate nuclear-polluted water,^51^ the application of the International Convention on the Law of the Sea, the 1972 London Convention, and the 1996 Protocol to the 1972 London Convention, international nuclear treaties, and transboundary harm do not offer a sufficiently robust basis for adjudicating uncertainties embedded in the discharge of tritium. If the adjudicative systems are ready to do boundary work between scientific uncertainties and policy approaches, then, on the one hand, the question would be what kind of risk assessment could be designed and in what way justified. On the other hand, how the “precautionary principle” could be institutionalized in international legal systems still remains controversial, given the various definitions possibly leading to arbitrary and capricious criteria (Pesendorfer, 2011, p. 287; Majone, 2002). Looking at the development of domestic, common law adjudications, a growing body of evidence associated with limitations of risk assessment, such as “the problem of mathematical proof” and “market share” liability for unidentified apportion rate, has been prevalent in adjudication, unlike international cases.

This study argues that the current international legal regime has institutional loopholes in responding to environmental disputes involving complex scientific uncertainties, as observed in the Fukushima contaminated water discharge case. Furthermore, domestic case laws in the U.S. and GMO disputes between Europe and the U.S. demonstrate that when scientific information, methodologies, and socio-political considerations are unclarified, a precautionary perspective can serve as a substantive standard for policymaking and judgment (Dupuy 2007). International disputes involving scientific uncertainties require institutionalized “inferential bridges” (National Research Council, 1983), an integrated judgmental mechanism that acknowledges uncertainty as a transitional prerequisite for adopting a new paradigm, rather than a prescriptive or quantitative risk-based measurement system. In brief, this research attends to the paradigm gap between science and law (i.e., observed reality *v*. constructive judgment) revealed by the precautionary approach, and introduces it as a leverage for the establishment of proactive governance, rather than reactive risk assessment or procedural formality. Specifically, international tribunals could adopt a reliability screening as a structured gatekeeping intervention (e.g., plausibility, irreversibility, proportionality, see Steel 2014) analogous to the Daubert framework to evaluate whether existing risk assessment methodologies adequately capture long-horizon and cumulative uncertainties that cannot be reduced to quantifiable standards or proof (IRGC 2005; Persson 2016; Renn 2026).

## Supplementary information

Supplementary information

## Data Availability

No datasets were generated or analysed during the current study.

## Appendix

### Appendix A

**Table:**

Annex C: Control, Inspection and Approval Procedures
1 Members shall ensure, with respect to any procedure to check and ensure the fulfillment of sanitary or phytosanitary measures, that:
(a) Such procedures are undertaken and completed without undue delay and in no less favorable manner for imported products than for like domestic products (emphasis added)

### Appendix B. Case-Law Analysis on Domestic Gatekeeping

This appendix presents the detailed case-law analysis referenced in (Risk assessment in domestic courts and gatekeeping on uncertainties). The material illustrates how U.S. domestic courts have institutionalized judicial gatekeeping over scientific uncertainty through evidentiary admissibility doctrines (Allen v. United States; Federal Rule of Evidence 702; Slatowski v. Sig Sauer) and tort causation rules (Sindell v. Abbott Laboratories).

As well stated in domestic law cases, regarding the difference between science and law, the Allen v. United States case indicates judicial flexibility first by commenting on the possibility of fallible “mathematical probability.” The “mathematical proof”^52^ ironically comes from the “arbitrary” selection of significance level by scientists (Allen v. United States 1984).^53^ The possibility that domestic courts can institutionalize gatekeeping on scientific uncertainty is not merely speculative. The 2023 amendment to Federal Rule of Evidence 702, the most significant revision since the Daubert decisions were codified into the Rule in 2000, reaffirmed the trial court’s obligation to determine, by a preponderance of the evidence, that experts meet each reliability requirement before presenting testimony to the jury.^54^ The amendment also made clear that expert opinions must stay within the reliable bounds of methodological validation.^55^ However, some circuits have continued to employ prior standards for case law after the amendment (Harvard Law Review 2025).^56^ This uneven pattern illustrates how the institutional variances of gatekeeping, not doctrinal text alone, determine which scientific uncertainties become legally visible and how flexibly and practically risk assessment can be adjudicated in courts.^57^

By vesting trial judges with the authority and obligation to evaluate the reliability of scientific methodology directly by incorporating the uncertainty argument, the Daubert framework enabled judges to actively construct what counts as fact in the courtroom by gatekeeping the pathway through which scientific knowledge becomes legally admissible. The governing question shifts from testing “general acceptance in the scientific community” to “methodological reliability for admissibility.” Courts are places of co-production that “bridge the gap between theory and reality.”^58^ In Slatowski v. Sig Sauer, Inc., 148 F.4th 132 (3 d Cir. 2025),^59^ the court clearly stated “the scientific method [is] the generation of testable hypotheses that are then subjected to the real-world crucible of experimentation, falsification/validation, and replication.” Also, the court reaffirmed that “competing expert opinions present the classic battle of the experts and it is up to a jury to evaluate what weight and credibility each expert opinion deserves.” (Phillips v. Cohen, 400 F.3 d 388, 399 (6th Cir. 2005) (internal quotation marks omitted)).^60^

The methodological fallacy of technological exclusion has long been recognized by many scholars. Traditionally, scientific rigor emanates from meticulous, numeric, quantifiable analysis, and standardized numbers have attempted to provide clear boundary work between the significance and insignificance of harm in scientific risk assessment. However, in courts, “causation here is a special legal construct.”^61^ The main issue in courts is less how to find facts than how to decide who should bear the costs of society’s inability to ascertain the relevant facts with any degree of certainty (Jasanoff, 1995: 123). In domestic mass tort law cases, “fact-finding, therefore, is a normative and deeply political exercise.” In Sindell v. Abbott Laboratories case, “market share” liability doctrine was applied to apportioning liability.^62^ Unlike decisions from the international adjudicative body, domestic law cases demonstrate more adaptive approaches to causation and “scientific indeterminacies” with a new jurisprudence: for instance, “cause in fact” and “legal cause.”^63^

### Appendix C. Extended Analysis on Marine Governance and Scientific Uncertainty

This appendix presents the extended analysis referenced in (The 2011 Fukushima Nuclear Disaster, contaminated water and the precautionary principle). It elaborates on the recent scientific literature on the discharge, the institutional normalization of uncertainty under the marine-governance regime, and the reasoning of the ITLOS Advisory Opinion in Case No. 31.

The biological effects of low-dose tritium exposure for a longer term remain unclear (Matsumoto et al., 2021). Furthermore, organically bound tritium may be accumulating in marine food chains through pathways not fully accounted for by current evaluation methods (Ferreira et al., 2024). The 30-year discharge schedule raises environmental concerns that the existing evaluation system is ill-equipped to address (Wang et al., 2024). Radioactive isotopes may also seep into coastal groundwater systems through seawater intrusion (Wei et al., 2024). While researchers have previously called for more comprehensive ocean monitoring (Richmond et al., 2022), no study has yet investigated the cumulative impact of the entire discharge period.

The Fukushima contaminated water discharge case has shown us that scientific uncertainties related to the long-term effects of radioactive contaminant discharges are often institutionally normalized within existing legal frameworks, and thus conflicts over uncertainty are amplified. Scientific uncertainties encompass the borderline nature of risk, which is often amplified and anchored in the fragmented structure, and further cemented by institutional deficiencies of the current international legal framework. While UNCLOS sets out general recommendations for risk assessment, ex-post reporting and mutual cooperation on marine discharges (Articles 205-207), the law does not set substantive legal thresholds of risk and harms associated with marine discharges, and therefore, within the current regime, the combination of unclear thresholds for disclosure of relevant data and methodologies, and the classification of polluting activities as land-based discharges rather than ‘dumping,’ means that risk and harms of discharges fall outside the scope of legal safeguards such as the London Protocol. As such, meeting the international concentration thresholds alone would disqualify the need to demonstrate long-term environmental impacts on local communities. The linear procedural requirements of monitoring, information sharing, and subsequent risk assessment and the avoidance of cumulative harm institutionally become *de facto* an externality within the system, intensifying social and psychological conflicts and instabilities (Jasanoff 1995). This is why risk assessments on the wastewater discharge should consider proactive measures, not reactive and passive ones.

Although the Advisory Opinion, Case No. 31^64^, addressed GHG-induced marine pollution rather than radionuclide discharge, its reasoning on uncertainty and precaution carries broader implications. The Advisory Opinion, Case No. 31, reinforces the analysis and the translation gap challenged in courts. Long-horizon ecological uncertainties remain complex to adjudicate, and procedural compliance cannot warrant the adequacy of legal control over scientific uncertainties. Attending to this, ITLOS redefined the scope of “pollution of the marine environment” (UNCLOS Article 1(1)(4))^65^. Furthermore, in ITLOS, Case No. 31 Advisory Opinion, paras. 212-213^66^, the Advisory Opinion rejected the coupling of scientific assessment and the institutional control over pollutant substances in environments and weighed in on “other relevant factors” that constitute “the best available science.” The following sections turn to WTO SPS jurisprudence^67^ and domestic gatekeeping as an operational comparison to construct inferential links across separate forums. How the institutionalized hurdles of the conventional methodological uncertainty framework, “risk assessment,” can complicate the legality of the “precautionary approach” as an alternative is central to the next sections. By implication, this reasoning substantiates the argument of “precautionary approach” to the uncertain potential, cumulative harms over a long duration caused by the discharge of radioactive wastewater from the Fukushima site. The following sections examine whether WTO SPS jurisprudence and domestic gatekeeping offer what marine governance cannot.

### Appendix D. Legal sources

#### Cases and advisory or arbitral decisions

Allen v. United States, 588 F. Supp. 247 (D. Utah 1984)

Chevron U.S.A., Inc. v. Natural Resources Defense Council, Inc., 467 U.S. 837 (1984)

Daubert v. Merrell Dow Pharmaceuticals, Inc., 509 U.S. 579 (1993)

Donaldson v. Central Illinois Public Service Co., 767 N.E.2 d 314 (Ill. 2002)

EC Measures Concerning Meat and Meat Products (Hormones), Appellate Body Report, WT/DS26/AB/R and WT/DS48/AB/R (adopted 13 February 1998)

European Communities—Measures Affecting the Approval and Marketing of Biotech Products (EC–Biotech), Panel Reports, WT/DS291/R, WT/DS292/R, WT/DS293/R (2006)

Frye v. United States, 293 F. 1013 (D.C. Cir. 1923)

In re Onglyza (Saxagliptin) and Kombiglyze (Saxagliptin and Metformin) Products Liability Litigation, No. 22-6078 (6th Cir. 2024)

Japan—Measures Affecting Agricultural Products (Japan–Agricultural Products), Appellate Body Report, WT/DS76/AB/R (1999)

Japan—Measures Affecting the Importation of Apples (Japan–Apples), Appellate Body Report, WT/DS245/AB/R (2003)

Loper Bright Enterprises v. Raimondo, 144 S. Ct. 2244 (2024)

Phillips v. Cohen, 400 F.3 d 388 (6th Cir. 2005)

Pulp Mills on the River Uruguay (Argentina v. Uruguay), Judgment, I.C.J. Reports 2010, p. 14

Request for an Advisory Opinion submitted by the Commission of Small Island States on Climate Change and International Law, Advisory Opinion, Case No. 31, ITLOS, 21 May 2024

Responsibilities and Obligations of States with Respect to Activities in the Area, Advisory Opinion, 1 February 2011, ITLOS Reports 2011, p. 10

Sindell v. Abbott Laboratories, 607 P.2 d 924 (Cal. 1980)

Slatowski v. Sig Sauer, Inc., 148 F.4th 132 (3 d Cir. 2025)

Soldo v. Sandoz Pharmaceuticals Corp., 244 F. Supp. 2 d 434 (W.D. Pa. 2003)

Trail Smelter Case (United States, Canada), Arbitral Awards, 3 UNRIAA 1905 (1938, 1941)

#### Treaties and international instruments

Agreement on the Application of Sanitary and Phytosanitary Measures (SPS Agreement) (1994) Marrakesh Agreement Establishing the World Trade Organization, Annex 1 A. United Nations Treaty Series 1867:493

Cartagena Protocol on Biosafety to the Convention on Biological Diversity (2000). Cartagena Protocol on Biosafety to the Convention on Biological Diversity, Montreal, 29 January 2000 (in force 2003). United Nations Treaty Series 2226:208

Convention on Biological Diversity (1992) Convention on Biological Diversity, Rio de Janeiro, 5 June 1992 (in force 1993). United Nations Treaty Series 1760:79

Convention on the Prevention of Marine Pollution by Dumping of Wastes and Other Matter (London Convention) (1972). Convention on the Prevention of Marine Pollution by Dumping of Wastes and Other Matter, London, 29 December 1972 (and the 1996 Protocol thereto). United Nations Treaty Series 1046:120

Rio Declaration on Environment and Development (1992). Rio Declaration on Environment and Development. United Nations *Report* A/CONF.151/26 (Vol. I)

United Nations Convention on the Law of the Sea (UNCLOS) (1982) United Nations Convention on the Law of the Sea, Montego Bay, 10 December 1982. United Nations Treaty *Series* 1833:397

Vienna Convention on the Law of Treaties (1969) Vienna Convention on the Law of Treaties, Vienna, 23 May 1969 (in force 1980). United Nations Treaty Series 1155:331

World Charter for Nature (1982) World Charter for Nature. United Nations General Assembly Resolution 37/7

#### Statutes, regulations, and rules

Directive 2001/18/EC (2001) Directive 2001/18/EC of the European Parliament and of the Council of 12 March 2001 on the deliberate release into the environment of genetically modified organisms. Official Journal of the European Communities L 106:1–39

Federal Rule of Evidence 702 (2023) Federal Rule of Evidence 702 (as amended Dec. 1, 2023). Legal Information Institute, Cornell Law School

Regulation (EC) No 1829/2003 (2003) Regulation (EC) No 1829/2003 of the European Parliament and of the Council of 22 September 2003 on genetically modified food and feed. Official Journal of the European Union L 268:1–23

TFEU (2016) Consolidated version of the Treaty on the Functioning of the European Union PART THREE: Union policies and internal actions—TITLE XX: Environment—Article 191 (ex Article 174 TEC). Official Journal of the European Union C 202:132–133
