# Supplementary material for: Precautionary Principle in Adjudicating Uncertainties: A Short Review on Science and Law
Source: Environ Manage. 2026 Jul 10;76(7):248. doi: 10.1007/s00267-026-02555-5 (PMC13350124; doi:10.1007/s00267-026-02555-5)
Supplement: Supplementary file 1 — Supplementary information [file 267_2026_2555_MOESM1_ESM.docx]

**Supplementary Information**

**Supplementary Notes**

The following notes correspond, in sequence, to the numbered superscripts in the main text and Appendices.

**Note 1.** Prior studies on exclusion in differentiating and closing the gap between scientific arguments and non-scientific communities, such as boundary work (Gieryn 1983), uncomfortable knowledge (Rayner 2012), and framing approach (Entman 1993), have emphasized how knowledge exclusion occurs (e.g., denial to know; interfacing science and policy; problem definitions), while paying less attention to the categorization of uncertainty characteristics. By clarifying these characteristics, the decisions in courts and legal tribunals can be more acutely tested and better understood for discussion.

**Note 2.** More broadly, “precautionary principle,” “sustainability reasoning,” “international environmental assessment,” “transboundary pollution,” “human rights approach,” and “environmental information and democracy” as procedural rights could be considered in adjudicating scientific uncertainties in courts (see Chen and Xu, 2022).

**Note 3.** “The facts that the law constructs (or reconstructs) are thus necessarily different from the facts that scientists construct to persuade their peers in their own rhetorically and procedurally distinctive surroundings.” (Jasanoff 1995, p. 10) (…) “Recognition that science is socially constructed in courtroom settings does not obviate the need for judges to decide in specific cases whose evidence should be admitted or how it should be weighted by the jury.” (Jasanoff 1995, p. 19)

**Note 4.** Doubts on adjudications, in worst cases, give rise to social backlash and discussions on the legitimacy, causing stress and burdens to judges, and further ignite the reformation of judicial systems (Schrever, Hulbert, and Sourdin 2024; Mauerhofer 2019). The political dynamics, compounded with the lack of a unified jurisdictional consensus, exacerbate the uncertainty of risk-related decisions in international disputes. For instance, when no legally binding authority for resolution over environmental uncertainties is designated, the fragmented interpretations of scientific data based on diverged jurisdictional boundaries may not converge in the international courtroom, and instead reinforce conflicts stemming from different procedures, evidentiary rules, and scientific experiments of risk assessment (see, e.g., Pulp Mills on the River Uruguay (Argentina v. Uruguay), Judgment, I.C.J. Reports 2010, p. 14; Kanhanga 2019; Shany, 2007).

**Note 5.** International Atomic Energy Agency (IAEA) (2022: 9). Uncertainties around untreated tritium are significant despite the positive result of task force investigation published in the IAEA report. “The taskforce noted that the REIA produced by TEPCO indicates that, using conservative assumptions (…) that was undertaken in the conduct of the REIA.”

**Note 6.** Kim (2022). According to Korean government, “the Japanese government announced that the water would undergo processing to ensure that most radioactive nuclides in it would remain below the allowable threshold for discharge, while untreated tritium would be diluted with seawater prior to its release.”

**Note 7.** As of 2022, groundwater and rainwater infiltrating the damaged buildings, together with the water used to cool the reactors, accumulated at the Fukushima Daiichi site at more than 150 tons of water per day (Buesseler et al. 2022). The contaminated water is gathered and accumulated on site in over 1,000 tanks, which is an emerging concern given the limited storage facilities. It is assumed approximately 1.25 million tons of contaminated water is stored, and only a quarter of tanks have been inspected regarding the concentration of radionuclides. Thus, how to dilute the water before the planned discharge is unclear.

**Note 8. “**TEPCO presented their approach for assessing uncertainties associated with the REIA. They identified the data, parameters and assumptions used in the REIA that were subject to uncertainty and the potential impact of these on the doses calculated to the representative person. TEPCO concluded that due to the choice of the source term and the conservative assumptions made, there is not any significant likelihood that the assessment results will exceed the dose constraint. (emphasis added)” (International Atomic Energy Agency (IAEA), 2022: 36).

**Note 9.** International Atomic Energy Agency (IAEA) (2022: 9); “very low and significantly below the dose constraint set by the regulatory body, NRA.” Also, the report stated “These findings provide confidence in TEPCO’s capability for undertaking accurate and precise measurements related to the discharge of ALPS treated water. Furthermore, based on the observations of the IAEA, TEPCO has demonstrated that it has a sustainable and robust analytical system in place to support the ongoing technical needs at FDNPS during the discharge of ALPS treated water.” (p. v).

**Note 10.** “However, as the source term is fundamental to the REIA, a more detailed and robust approach was suggested by the Task Force for improving the understanding of interested parties and enhancing transparency (IAEA, 2022, p. 20)” “The Task Force welcomed the plans for enhanced environmental monitoring and stressed that high quality, representative monitoring programmes – both source and environmental – for assessing the potential impact of discharges of ALPS treated water on people and the environment are a requirement for regulatory control. Furthermore, the Task Force emphasized that demonstrably high-quality data – sufficiently accurate and precise – are vital for facilitating transparency and communicating with relevant stakeholders (IAEA, 2022, p. 45).”

**Note 11.** No regulation of the 1972 London Convention and the 1996 Protocol to the 1972 London Convention applies to the discharge of wastewater from the site into the sea. *See*, Takamura (2014)

**Note 12.** A different interpretation is the intentional disposal of waste should be subject to the provision of ‘the fullest possible extent’ in UNCLOS (Articles 194(3)(a)).

United Nations Convention on the Law of the Sea Article 194(3)(a)3.

The measures taken pursuant to this Part shall deal with all sources of pollution of the marine environment. These measures shall include, inter alia, those designed to minimize to the fullest possible extent: (a)  the release of toxic, harmful or noxious substances, especially those which are persistent, from land-based sources, from or through the atmosphere or by dumping;

**Note 13.** Takamura (2014: 96). “Presently, no international nuclear treaty has provisions specifically prohibiting and regulating the release of radioactive materials into the sea as was experienced by the Fukushima Daiichi NPP case.”

**Note 14.** The Trail Smelter case confirmed there is no state entitled to cause transboundary damage. However, in the Japanese discharge case, the harm is not evident and hard to prove (Trail Smelter Case, 3 UNRIAA 1905, 1938 and 1941). This is a limitation of common law as a retrospective measure.

**Note 15.** UNCLOS, article 207(3).

**Note 16.** In addition, according to UNCLOS article 200, states shall “endeavor to participate actively in regional and global programmes to acquire knowledge for the assessment of the nature and extent of pollution, exposure to it, and its pathways, risks and remedies.”

**Note 17.** *See*, The Government of Japan (2021). The wastewater falls within the safety boundary and meets the safety standard. The tritium concentration is 1/40 of the Japanese standard (60,000Bq/L) and 1/7 of WHO drinking water guidelines (10,000 Bq/L). *See* also, Chen and Xu (2022).

**Note 18.** Matsumoto et al. (2021): “One should recognize the relationship between the dose of tritium exposure and the resulting risk based on scientific evidence. However, the biological effects of low dose or low-dose rate radiation remain controversial regardless of the types of radiations.”

**Note 19.** Loper Bright Enterprises v. Raimondo, 144 S. Ct. 2244 (2024).

**Note 20.** Chevron U.S.A., Inc. v. Natural Resources Defense Council, Inc., 467 U.S. 837 (1984).

**Note 21.** For understanding GMO product uncertainties, *see*, Myhr (2010). In this paper, the author emphasizes “post-normal science” approaches by Funtowicz and Ravetz (1991). “Scientific understanding of the factor affecting health and environmental risks is still developing and it is unlikely that controversies over the risks of GM crops will be resolved in the near future. For instance, a non-uniform response is seen among experts to new studies reporting deviations from safety assumptions. The study by Quist and Chapela (2001), reporting unexpected introgression of genes from maize into maize landraces in Mexico was highly controversial. A more recent example is the study by Rosi-Marshall et al. (2007), which found the toxins from Bt maize by-products and litter could be found in headwater streams in Midwest USA. They also reported that they had in laboratory studies detected reduced growth among aquatic trichopterans feeding Bt-maize litter compared to non-GM litter”; For “post-normal science,” *see*, (Funtowicz and Ravetz, 1991: 145).

**Note 22.** Many scholars differentiated “insufficient information” with “scientific uncertainties.” See, Henckels (2006).

**Note 23.** WTO (2006: 1073), “3. EC member State safeguard measures.”

**Note 24.** “Risk Assessment” is defined by SPS Agreement Annex A(4): “Risk assessment — The evaluation of the likelihood of entry, establishment or spread of a pest or disease within the territory of an importing Member according to the sanitary or phytosanitary measures which might be applied, and of the associated potential biological and economic consequences; or the evaluation of the potential for adverse effects on human or animal health arising from the presence of additives, contaminants, toxins or disease-causing organisms in food, beverages or feedstuffs.”

**Note 25.** The WTO panel report stated that:

“The relevant EC scientific committee subsequently also reviewed the arguments and the evidence submitted by the member State to justify the prohibition and did not consider that such information called into question its earlier conclusions. The Panel thus considered that sufficient scientific evidence was available to permit a risk assessment as required by the SPS Agreement. Hence, in no case was the situation one in which the Panel had been persuaded that the relevant scientific evidence was insufficient to perform a risk assessment, such that the member State might have had recourse to a provisional measure under Article 5.7 of the SPS Agreement.” (WTO, 2006: 8.9).

**Note 26.** Article 5.1 (Assessment of Risk and Determination of the Appropriate Level of Sanitary or Phytosanitary Protection) states that:

“Members shall ensure that their sanitary or phytosanitary measures are based on an assessment, as appropriate to the circumstances, of the risks to human, animal or plant life or health, taking into account risk assessment techniques developed by the relevant international organizations.”

**Note 27.** SPS Agreement Annex A3(a):

“for food safety, the standards, guidelines and recommendations established by the Codex Alimentarius Commission relating to food additives, veterinary drug and pesticide residues, contaminants, methods of analysis and sampling, and codes and guidelines of hygienic practice.” (emphasis added)

**Note 28.** Article 5.7 states:

“In cases where relevant scientific evidence is insufficient, a Member may provisionally adopt sanitary or phytosanitary measures on the basis of available pertinent information, including that from the relevant international organizations as well as from sanitary or phytosanitary measures applied by other Members. In such circumstances, Members shall seek to obtain the additional information necessary for a more objective assessment of risk and review the sanitary or phytosanitary measure accordingly within a reasonable period of time.”;

Also, in Japan – Apples (WT/DS245/AB/R) para. 179, “(…) The first requirement of Article 5.7 is that there must be insufficient scientific evidence. When a panel reviews a measure claimed by a Member to be provisional, that panel must assess whether "relevant scientific evidence is insufficient (…) Article 5.1 sets out a key discipline under Article 5, namely that "Members shall ensure that their sanitary or phytosanitary measures are based on an assessment … of the risks to human, animal or plant life or health" (…)”

**Note 29.** WTO (2006: 8.9); According to the Panel, the “Panel examined likewise whether the risk assessment undertaken by the EC scientific committees could provide reasonable support for a prohibition of the biotech products at issue, but considered that this was not the case.” (WTO, 2006: 8.10).

**Note 30.** “The Appellate Body’s jurisprudence on art 5.7 has varied in the emphasis it places on the role of a precautionary approach.” Henckels (2006: 294); In the case of *EC-Hormones,* the Appellate Body stated that “(…) We agree, at the same time, with the European Communities, that there is no need to assume that Article 5.7 exhausts the relevance of a precautionary principle. It is reflected also in the sixth paragraph of the preamble and in Article 3.3 …” (*See*, Laowonsiri (2010: 565, 577); See also, WTO (2006: 7.3065, note 1095, 3971) “the precautionary principle ‘has not been written into the SPS Agreement as a ground for justifying SPS measures that are otherwise inconsistent with the obligations of Members set out in particular provisions of that Agreement’.”

**Note 31.** WTO (2006: 971). “However, even if a Member follows a precautionary approach, its SPS measures need to be ‘based on’ (i.e., ‘sufficiently warranted’ or ‘reasonably supported’ by) a risk assessment. Or, to put it another way, such an approach needs to be applied in a manner consistent with the requirements of Article 5.1.”

**Note 32.** “7.78 The European Communities asserts that the precautionary principle has by now become a fully-fledged and general principle of international law. According to the European Communities, the precautionary principle was first recognised in the World Charter for Nature, adopted by the UN General Assembly in 1982, and was subsequently incorporated into various international conventions on the protection of the environment.” WTO (2006: 336); “The United States argues that the European Communities has not identified how a ‘precautionary principle’ would be of relevance to interpreting any particular provision of the WTO Agreement” (WTO 2006; 337).

**Note 33.** The Rio Declaration on Environment and Development (1992) Principle 15 stated, “In order to protect the environment, the precautionary approach shall be widely applied by States according to their capabilities. Where there are threats of serious or irreversible damage, lack of full scientific certainty shall not be used as a reason for postponing cost-effective measures to prevent environmental degradation.”

In contrast, in EC Measures Concerning Meat and Meat Products (Hormones), Appellate Body Report, WT/DS26/AB/R, WT/DS48/AB/R, adopted 16 January 1998, para. 121:

“The basic submission of the European Communities is that the precautionary principle is, or has become, 'a general customary rule of international law' or at least 'a general principle of law'. Referring more specifically to Articles 5.1 and 5.2 of the SPS Agreement, applying the precautionary principle means, in the view of the European Communities, that it is not necessary for *all* scientists around the world to agree on the “possibility and magnitude” of the risk, (…).”

**Note 34.** The economic cost concern can also be alleviated given the balancing between irreversible harm and upfront cost of precautionary measures Shaw and Schwartz (2005: 5).

**Note 35.** Cartagena Protocol on Biosafety to the Convention on Biological Diversity is an international agreement, which was adopted on Jan. 29^th^, 2000, and became effective on Sep. 11^th^, 2003. Thus far, 173 countries have ratified the protocol, but the US has not signed nor ratified the protocol. The issue is that US does not sign or ratify the Cartagena Protocol, which states domestic regulation and risk assessment. Thus, the SPS agreement precedes this Cartagena Protocol and therefore there was no appropriate risk assessment according to the SPS agreement.

**Note 36.** “The Panel also considered whether any risk assessment had been provided by the relevant member States which would reasonably support the prohibition of the biotech products at issue. Although some of the member States did provide scientific studies, in no case did they provide an assessment of the risks to human health and/or the environment meeting the requirements of the SPS *Agreement*. The Panel likewise examined whether the risk assessments undertaken by the EC scientific committees could provide reasonable support for a prohibition of the biotech products at issue but considered that this was not the case. In the light of this, the Panel has concluded that each of the safeguard measures taken by the relevant member States fails to meet the obligations of the European Communities under the SPS *Agreement*.” WTO (2006: 8.10).

**Note 37.** And “such risk assessment shall be based, at a minimum, on information provided in accordance with Article 8 and other available scientific evidence in order to identify and evaluate the possible adverse effects of living modified organisms on the conservation and sustainable use of biological diversity, taking also into account risks to human health.” The article 15 of Cartagena Protocol on Biosafety to the Convention on Biological Diversity.

**Note 38.** Directive 2001/18/EC of the European Parliament and of the Council on the deliberate release into the environment of genetically modified organisms and repealing Council Directive 90/220/EEC.

**Note 39.** Article 1 (Objective) “In accordance with the precautionary principle, the objective of this Directive is to approximate the laws, regulations and administrative provisions of the Member States and to protect human health and the environment when: carrying out the deliberate release into the environment of genetically modified organisms for any other purposes than placing on the market within the Community; placing on the market genetically modified organisms as or in products within the Community.”

**Note 40.** As stated in Article 191(2) of Treaty on the Functioning of the European Union [TFEU], 2016 stipulates that environmental policies should be based on the precautionary principle.

**“**2. Union policy on the environment shall aim at a high level of protection taking into account the diversity of situations in the various regions of the Union. It shall be based on the precautionary principle and on the principles that preventive action should be taken, that environmental damage should as a priority be rectified at source and that the polluter should pay. (…)”

**Note 41.** Regulation (EC) No 1829/2003 of the European Parliament and of the Council of 22 September 2003. If the qualitative characteristic of risk (e.g., value) is admitted and employed in defining risk assessment, “community procedures” conforming to local governance and environments rather than unified standard developed by international organizations would be also persuasive.

**Note 42.** Trouwborst 2009, p. 108 “Already by the early 1990s, the application of the principle by States had become so widespread and consistent that the customary international law question came into play. An analysis carried out a decade later, testing the relevant conduct and statements of States against the generally accepted standards on the formation of customary or general international law, and taking account also of international jurisprudence and doctrine, prompted the conclusion that the core content of the precautionary principle had by then indeed attained the status of customary international law.”

**Note 43.** Hansson (2020), p. 249, “Practical decision-making that complies with these principles can be said to follow the tenets of science-based precaution. It can be seen from the official documents referred to above that this model corresponds closely to what is officially meant by the precautionary principle. In particular, public authorities endorsing the precautionary principle emphasize that it can only be triggered by scientifically valid indications of danger. For instance, the Swedish Chemicals Legislation from 1985 required a “reasonable scientific foundation” in order to trigger precautionary measures. (Hansson, 2020, p. 249)

**Note 44.** Gilland (2000). Precaution, GM crops and farmland birds. In J. Morris (Ed.), Rethinking Risk and the Precautionary Principle (pp. 60-83). Oxford: Butterworth-Heinemann.

**Note 45.** Regarding loss aversion, biased heuristics, please refer to Sunstein (2003): “The precautionary principle often becomes operational only because of loss aversion, as people take precautions against potential losses from the status quo, but neglect potential benefits that would be unmistakable gains.” (p. 8)

**Note 46.** In EC-Hormones (WT/DS26/AB/R, para. 122), “The United States does not consider that the "precautionary principle" represents customary international law and suggests it is more an "approach" than a "principle". Canada, too, takes the view that the precautionary principle has not yet been incorporated into the corpus of public international law; however, it concedes that the "precautionary approach" or "concept" is "an emerging principle of law" which may in the future crystallize into one of the "general principles of law recognized by civilized nations" within the meaning of Article 38(1)(c) of the *Statute of the International Court of Justice*.” (emphasis added);

**Note 47.** The gaps between realities and the availability of data in scientific theories and modeling contribute to gaps in scientific methods of risk assessment. In this regard, “the judgments made by the scientist/risk assessor for each component of risk assessment” intervene in optional policies for risk assessment as well (National Research Council, 1983: 28)

**Note 48.** Traditional reductionism science equates the truth of a hypothesis with the proof of predictions inferred from this hypothesis (Toulmin, 1961: 24). If a scientific risk modeling produces a predicted outcome that is congruent with observed (empirical) data, the hypotheses of this scientific modeling could be verified and confirmed (Oreskes et al. 1994: 643). However, this reductionist view on scientific predictions and verification logic for generalization includes innate logical fallacies (Oreskes et al. 1994: 642-643). The limitation of reductionist generality turns out to be more evident in these complex problems. For instance, solutions to acidification problems should examine multiple interdependent social variables due to the open-system characteristic of society (Oreskes et al. 1994: 645). This is why the enhancement of forecast accuracy of risk assessment does not directly give rise to rational results (Pielke 1999: 90-93; Sarewitz and Pielke 1999)

**Note 49.** Renn et al., 2004: "If a lack of scientific certainty has been identified in screening, then the subsequent regulatory process takes the form of precautionary appraisal. This involves a broad-based approach, with the full engagement of different interested and affected parties and which does not rely on probabilistic techniques." (Renn et al. 2004, p. 8); also, "If uncertainty plays a large role, in particular ignorance, the risk-based approach becomes counter-productive. Judging the relative severity of risks on the basis of uncertain parameters does not make much sense. Under these circumstances, management strategies belonging to the precautionary approach are required." (Renn et al. 2004, p. 10)

**Note 50.** The quantifiable variables, such as the probability of an accident and the consequences of lost money or deaths, are the main components of a formula for risk assessment.

**Note 51.** The international assessment and collaboration of exchange of information governed by UN Convention on the Law of the Sea are not the viable options to accept uncertainties.

**Note 52.** Allen v. United States, 588 F. Supp. 247, 416 (D. Utah 1984).

**Note 53.** As stated in Allen v. United States, 588 F. Supp. 247, 416; 418 (D. Utah 1984)

**“**Where p = 0.01 or 0.001, the probability that the observed correlation resulted from random chance is 1 in 100, or 1 in 1,000, respectively. Whether a statistical increase or relationship is "significant" depends first upon what arbitrary level of significance a researcher has selected in analyzing the data. A researcher selecting an arbitrary level p = 0.05 has determined that where the probability is 1 in 20 that something resulted from random chance (and, conversely, that the probability is 19 out of 20 that it did not result from chance), the relationship will be deemed "significant"; where, for instance, the probability is 1 in 19 that events happened by chance (p = 0.0526), the relationship will be deemed statistically "insignificant" even though the probability is 94.73% or 18 chances out of 19 that the observed relationship is not a random event. Though deemed "insignificant" by the researcher, the certainty that the observed increase is related to its hypothetical cause rather than mere chance is still far more likely than not (…)” (p. 416)

“The value of the available statistical data concerning radiation and cancer in off-site communities is not confined by arbitrary tests of "statistical significance." Nor is the court constrained by simplistic models of causal probability impressed upon the judicial "preponderance of the evidence" standard.” (p. 418)

**Note 54.** Fed. R. Evid. 702. (2023) Rule 702. Testimony by Expert Witnesses

“A witness who is qualified as an expert by knowledge, skill, experience, training, or education may testify in the form of an opinion or otherwise if the proponent demonstrates to the court that it is more likely than not that: (a) the expert’s scientific, technical, or other specialized knowledge will help the trier of fact to understand the evidence or to determine a fact in issue; (b) the testimony is based on sufficient facts or data; (c) the testimony is the product of reliable principles and methods; and (d) the expert's opinion reflects a reliable application of the principles and methods to the facts of the case.” (As amended Apr. 17, 2000, eff. Dec. 1, 2000; Apr. 26, 2011, eff. Dec.1, 2011; Apr. 24, 2023, eff. Dec. 1, 2023.)

**Note 55.** See Fed. R. Evid. 702 (as amended Dec. 1, 2023); Fed. R. Evid. 702 advisory committee’s note for details:

“Rule 702(d) has also been amended to emphasize that each expert opinion must stay within the bounds of what can be concluded from a reliable application of the expert’s basis and methodology. Judicial gatekeeping is essential because just as jurors may be unable, due to lack of specialized knowledge, to evaluate meaningfully the reliability of scientific and other methods underlying expert opinion, jurors may also lack the specialized knowledge to determine whether the conclusions of an expert go beyond what the expert’s basis and methodology may reliably support.”

The 2023 amendment to Rule 702 reaffirmed the trial court’s gatekeeping obligation and clarified the judicial governance of knowledge co-production by requiring that expert testimony be established by a preponderance of the evidence and that expert opinions remain within the reliable bounds of the underlying methodology.

**Note 56.** “In a study conducted by the Lawyers for Civil Justice, sixty-one percent of federal courts were divided about whether the preponderance of the evidence standard applied to the admissibility of expert testimony.” (Harvard Law Review 2025); See Bush and Kuhn 2023, pp. 54-55.

**Note 57.** For a comprehensive discussion of early post-amendment case law, see advisory committee’s notes to Federal Rule of Evidence 702 and also Harvard Law Review 2025; Mark A. Behrens & Andrew J. Trask, Federal Rule of Evidence 702: A History and Guide to the 2023 Amendments Governing Expert Evidence, 12 Tex. A&M L. Rev. 43, 44–45 (2024).

**Note 58.** *Slatowski v. Sig Sauer, Inc.*, 148 F.4th 132 at 10 (3d Cir. 2025).

**Note 59.** *Slatowski v. Sig Sauer, Inc.*, 148 F.4th 132 at 10 (3d Cir. 2025):

“That is a problem for Slatowski. Expert testimony offered in federal court must be “the product of reliable principles and methods.” Fed. R. Evid. 702. “The hallmark of Daubert’s reliability prong is the scientific method.” Soldo v. Sandoz Pharms. Corp., 244 F. Supp. 2d 434, 559 (W.D. Pa. 2003) (explaining Daubert v. Merrell Dow Corp., 509 U.S. 579, 593 (1992)). In turn, “the scientific method [is] the generation of testable hypotheses that are then subjected to the real-world crucible of experimentation, falsification/validation, and replication.” Id. at 457; see also Daubert, 509 U.S. at 590, 593 (requiring “appropriate validation” and focusing on “whether [a theory or technique] can be (and has been) tested”). Speculation does not cut it. After all, “the word ‘knowledge’ connotes more than subjective belief or unsupported speculation.” Daubert, 509 U.S. at 590. Yet Slatowski never bridges the gap between theory and reality. Instead, he just touts his theory. His experts explained how easily P320s fire, reviewed statistics about how often other P320s fire accidentally, and analyzed why those accidents might be so common. But neither expert considered how the conditions that could cause accidental firing might have manifested on the day of Slatowski’s accident. Their testimony is reliable about whether the P320’s design *could have* caused an accident, but not whether it *did* cause this accident. That would have required not just theory, but factual context.”

**Note 60.** In re Onglyza (Saxagliptin) and Kombiglyze (Saxagliptin and Metformin) Products Liability Litigation, No. 22-6078, slip op. at 10 (6th Cir. 2024); Also, at 8, “But the court cautioned that epidemiology studies only allow “experts to find associations, which by themselves do not entail causation”—experts must still do “the work to bridge the gap between association and causation.” Id. (internal quotation marks omitted).”

**Note 61.** Jasanoff (1995: 119); In the book, Jasanoff examined the relationship between “scientific indeterminacies” and policy consideration (Jasanoff 1995: 122).

**Note 62.** Jasanoff (1995: 123); Sindell v. Abbott Laboratories, 607 P.2d 924 (1980). “Under this approach, each manufacturer's liability would approximate its responsibility for the injuries caused by its own products. Some minor discrepancy in the correlation between market share and liability is inevitable; therefore, a defendant may be held liable for a somewhat different percentage of the damage than its share of the appropriate market would justify. It is probably impossible, with the passage of time, to determine market share with mathematical exactitude. Sindell, at 937. Note that the ‘market share liability’ doctrine is not adopted universally and remains contestable.

**Note 63.** For instance, in Donaldson v. Central Illinois Public Service Co., 767 N. E. 2d 314 (Ill. Sup. Ct. 2002).

**Note 64.** *Request for an Advisory Opinion submitted by the Commission of Small Island States on Climate Change and International Law*, Advisory Opinion, Case No. 31, International Tribunal for the Law of the Sea, 21 May 2024.

**Note 65.** UNCLOS Article 1(1)(4)) "pollution of the marine environment" means the introduction by man, directly or indirectly, of substances or energy into the marine environment, including estuaries, which results or is likely to result in such deleterious effects as harm to living resources and marine life, hazards to human health, hindrance to marine activities, including fishing and other legitimate uses of the sea, impairment of quality for use of sea water and reduction of amenities;”

**Note 66.** ITLOS, Case No. 31 Advisory Opinion, paras. 212–213.

Para. 212. The Tribunal considers that in the determination of necessary measures to prevent, reduce and control marine pollution from anthropogenic GHG emissions, the science undoubtedly plays a crucial role, as it is key to understanding the causes, effects and dynamics of such pollution and thus to providing the effective response. However, this does not mean that the science alone should determine the content of necessary measures. In the Tribunal’s view, as indicated above, there are other relevant factors that should be considered and weighed together with the best available science.

Para. 213. The Tribunal wishes to add at this juncture that in determining necessary measures, scientific certainty is not required. In the absence of such certainty, States must apply the precautionary approach in regulating marine pollution from anthropogenic GHGs. While the precautionary approach is not explicitly referred to in the Convention, such approach is implicit in the very notion of pollution of the marine environment, which encompasses potential deleterious effects. In this regard, the Tribunal recalls the observation of the Seabed Disputes Chamber in Responsibilities and Obligations of States with Respect to Activities in the Area (hereinafter “the Area Advisory Opinion”) that the precautionary approach has been incorporated into a growing number of international treaties and other instruments, many of which reflect the formulation of Principle 15 of the Rio Declaration. In the view of the Chamber, this has initiated a trend towards making this approach part of customary international law. (Responsibilities and obligations of States with respect to activities in the Area, Advisory Opinion, 1 February 2011, ITLOS Reports 2011, p. 10, at p. 47, para. 135) For marine pollution arising from anthropogenic GHG emissions, the precautionary approach is all the more necessary given the serious and irreversible damage that may be caused to the marine environment by such pollution, as is assessed by the best available science.

**Note 67.** Agreement on the Application of Sanitary and Phytosanitary Measures arts. 2.2, 5.1, 5.7; World Trade Organization, *WTO Analytical Index: SPS Agreement—Article 5*.
